# Daxx Inhibits HIV-1 Reverse Transcription and Uncoating in a SUMO-Dependent Manner

**DOI:** 10.3390/v12060636

**Published:** 2020-06-11

**Authors:** Sarah Maillet, Juliette Fernandez, Mathilde Decourcelle, Khadija El Koulali, Fabien P. Blanchet, Nathalie J. Arhel, Ghizlane Maarifi, Sébastien Nisole

**Affiliations:** 1 Institut de Recherche en Infectiologie de Montpellier (IRIM), Université de Montpellier, CNRS, 34090 Montpellier, France; sarah.maillet@irim.cnrs.fr (S.M.); juliette.fernandez@irim.cnrs.fr (J.F.); fabien.blanchet@irim.cnrs.fr (F.P.B.); nathalie.arhel@irim.cnrs.fr (N.J.A.); ghizlane.maarifi@irim.cnrs.fr (G.M.); 2 BCM, Université de Montpellier, CNRS, INSERM, 34090 Montpellier, France; Mathilde.Decourcelle@fpp.cnrs.fr (M.D.); khadija.el-koulali@fpp.cnrs.fr (K.E.K.)

**Keywords:** Daxx, HIV-1, reverse transcription, uncoating, restriction factor, interferon, ISG, intrinsic immunity, host defense

## Abstract

Death domain-associated protein 6 (Daxx) is a multifunctional, ubiquitously expressed and highly conserved chaperone protein involved in numerous cellular processes, including apoptosis, transcriptional repression, and carcinogenesis. In 2015, we identified Daxx as an antiretroviral factor that interfered with HIV-1 replication by inhibiting the reverse transcription step. In the present study, we sought to unravel the molecular mechanism of Daxx-mediated restriction and, in particular, to identify the protein(s) that Daxx targets in order to achieve its antiviral activity. First, we show that the SUMO-interacting motif (SIM) located at the C-terminus of the protein is strictly required for Daxx to inhibit HIV-1 reverse transcription. By performing a quantitative proteomic screen combined with classical biochemical analyses, we found that Daxx associated with incoming HIV-1 cores through a SIM-dependent interaction with cyclophilin A (CypA) and capsid (CA). Daxx was found to reside within a multiprotein complex associated with viral capsids, also containing TNPO3, TRIM5α, and TRIM34. Given the well-known influence of these cellular factors on the stability of HIV-1 cores, we investigated the effect of Daxx on the cytoplasmic fate of incoming cores and found that Daxx prevented HIV-1 uncoating in a SIM-dependent manner. Altogether, our findings suggest that, by recruiting TNPO3, TRIM5α, and TRIM34 and possibly other proteins onto incoming HIV-1 cores through a SIM-dependent interaction with CA-bound CypA, Daxx increases their stability, thus preventing uncoating and reverse transcription. Our study uncovers a previously unknown function of Daxx in the early steps of HIV-1 infection and further illustrates how reverse transcription and uncoating are two tightly interdependent processes.

## 1. Introduction

HIV-1, like all viruses, is an obligate parasite and requires numerous cellular factors and pathways to replicate. As a result, mammalian cells have developed specific factors that function in a cell-autonomous manner to limit virus replication. These have been termed restriction factors and constitute the so-called intrinsic immunity, which is now considered to be the third arm of immunity [1]. Most restriction factors, however, even if they are constitutively expressed, are upregulated by type I interferon(s), and thus intrinsic defenses can be considered to be a part of antiviral innate immunity [2,3]. Since the discovery of the first HIV-1 restriction factor, in 2002, APOBEC3G [4], many other factors have been identified and shown to inhibit nearly all steps of the HIV-1 replicative cycle (reviewed in [5,6,7]). However, reverse transcription appears to be a hotspot for restriction, since it is targeted by several potent antiviral factors, including TRIM5α [8], APOBEC3G [4,9], and SAMHD1 [10,11]. Reverse transcription of HIV-1 genomic RNA occurs within incoming cores that are released in the cytosol following viral entry and undergo morphological changes including disassembly, which is referred to as uncoating (reviewed in [12,13]). Although the mechanisms underlying the spatial and temporal regulation of reverse transcription and uncoating are still under debate, evidence is emerging that they are tightly linked and coordinated. Indeed, while TRIM5α has been shown to inhibit reverse transcription through a premature disassembly of viral cores [14], inhibition of reverse transcription delayed uncoating [15,16,17], and reverse transcription itself was shown to initiate capsid disassembly [18,19]. A proper coordination of reverse transcription and uncoating is essential to ensure a productive infection. The coordination of this chain of events that ultimately leads to nuclear import and integration involves several viral and cellular proteins (reviewed in [12,20,21]). Among the numerous cellular factors that interact with HIV-1 cores and control their stability are cyclophilin A (CypA) [22], TNPO1 [23], TNPO3 [24,25], Nup153 [26], TRIM34 [27,28], Pin1 [29], and CPSF6 [30] (reviewed in [31]). CypA binds to the so-called CypA-binding loop in the N-terminal domain of the capsid (CA) protein and is incorporated inside viral particles [32,33]. Despite intensive research, however, its exact role in the early steps of HIV-1 infection has remained elusive. Disruption of CypA–CA interactions by either treatment with cyclosporine or its analogues (e.g., Debio 025) or by mutation within CA (e.g., G89V or P90A) decreased HIV-1 infection, by affecting uncoating or reverse transcription in certain cell types [34,35,36,37]. CypA has also been recently found to protect HIV-1 from TRIM5α-mediated restriction in human primary cells [38,39].

In 2015, we identified the death domain-associated protein 6 (Daxx) as a new antiviral factor interfering with HIV-1 replication and showed that reverse transcription was the step being affected [40]. Although we showed that restriction occurred at the step of reverse transcription, the mechanism involved was unclear. Daxx is a multifunctional, ubiquitously expressed and highly conserved protein through evolution. It has no enzymatic activity, but rather acts as a scaffold to bridge different proteins, thus regulating their function (reviewed in [41,42,43]). Although this multifunctional adapter is present in the cytoplasm, it is mainly expressed in the nucleus, where it localizes within promyelocytic leukemia protein (PML) nuclear bodies. The recruitment of Daxx within PML nuclear bodies is mediated by its direct interaction with SUMOylated PML through the SUMO-interacting motif (SIM) located at its C-terminal end [44,45]. Although it is itself SUMOylated, most of Daxx functions are mediated by its SIM, which binds and recruits SUMO-conjugated proteins. Daxx has been implicated in several biological processes, but its implication in apoptosis and transcriptional repression have been particularly well characterized (reviewed in [41,42,43]). It is interesting to note that Daxx has been shown to restrict several viruses, mostly DNA viruses, but also retroviruses, through its capacity to repress transcription [42,46,47,48,49].

In the present study, we investigated how Daxx inhibits HIV-1 reverse transcription. First, we determined that Daxx expression is upregulated by interferon (IFN) in both murine and human cells. Furthermore, we showed that Daxx is a mediator of the type I IFN-induced inhibition of HIV-1 reverse transcription. Next, by using Daxx mutants, we established that the SIM domain was required for the Daxx-mediated inhibition of reverse transcription, thus suggesting that Daxx’s target, be it viral or cellular, was SUMOylated. In order to identify this target, we performed a proteomic screen of cellular proteins interacting with Daxx, using stable-isotope labeling by amino acids in cell culture (SILAC) coupled with liquid chromatography tandem mass spectrometry (LC-MS/MS). This screen identified several previously-identified HIV-1 core-binding proteins, including CypA. Further biochemical analyses revealed that Daxx binds to incoming HIV-1 cores through a SIM-dependent interaction with CypA. Furthermore, we found that Daxx recruits several proteins onto viral capsids, including TNPO3, TRIM5α, and TRIM34, which is concordant with its role as a scaffolding protein. Finally, we performed fate-of-capsid assays to demonstrate that Daxx inhibits the uncoating of incoming HIV-1 cores. Altogether, our results suggest that, by binding to CypA through its SIM domain, Daxx forms a multiprotein complex around HIV-1 cores that prevents uncoating, and therefore inhibits reverse transcription.

## 2. Materials and Methods

### 2.1. Plasmids

Plasmids constructs expressing HA-tagged Daxx wild-type (wt), and 1-732 and 15KR Daxx mutants were kindly provided by Hsiu-Ming Shih (Institute of Biomedical Sciences, Academia Sinica, Taipei, Taiwan) [44]. The plasmid expressing Flag-tagged human TRIM5α was provided by Stephen P. Goff (Columbia University, New York, NY, USA) [50].

### 2.2. Cell Culture and Transfections

HeLa cells, HEK293T, and MDTF cells were purchased from the American Type Culture Collection (ATCC, Manassas, VA, USA) and cultured at 37 °C and 5% CO_2_ in DMEM containing 10% FCS, supplemented with 1% penicillin-streptomycin (Thermo Fisher Scientific, Waltham, MA, USA). Transient plasmid transfections were performed using either Lipofectamine 2000 (Thermo Fisher Scientific) for HEK293T cells, or FuGENE 6 (Promega, Madison, WI, USA) for HeLa cells, following the manufacturer’s instructions. Daxx-specific and untargeting (CTR) siRNA were purchased from Dharmacon (Lafayette, CO, USA) as pools of 4 individual siRNA (ON-TARGETplus SMARTpool). Transfections of siRNA were performed using HiPerFect Transfection Reagent (Qiagen, Hilden, Germany), according to the manufacturer’s instructions.

### 2.3. Viruses and Vectors

Defective-interfering H4 Sendai virus (SeV) was provided by Dominique Garcin (Department of Microbiology and Molecular Medicine, University of Geneva, Geneva, Switzerland) and was used at 50 HAU/mL [51]. HIV-1 vectors encoding GFP were produced by transient cotransfection of HEK293T cells using calcium-phosphate precipitation with a GFP-expressing HIV-1 vector (pCSGW), an encapsidation plasmid (p8.91 for HIV-1 wt, or the corresponding capsid mutant P90A), and a VSV-G envelope expression plasmid (pVSVG). The vectors were harvested after 2 days, filtered and ultracentrifuged at 22,000 rpm for 1 h, at 4 °C (SW32Ti rotor), and titred by flow cytometry measuring the GFP expression.

### 2.4. Antibodies and Reagents

For the detection of Daxx in human cells by Western blot and immunofluorescence, we used a Daxx monoclonal antibody (clone 7A11) purchased from Abnova (Taipei City, Taiwan). For detecting murine Daxx by Western blot, the monoclonal anti-Daxx antibody (clone H-7) from Santa Cruz Biotechnology (Dallas, TX, USA) (sc-8043) was used. Other primary antibodies used in Western blot and immunoprecipitation experiments were the following: mouse monoclonals against β-actin and FLAG (from Merck KGaA, Darmstadt, Germany), mouse anti-p24 (NIH AIDS Reagent program clone 183-H12-5C), mouse anti-TNPO1 (Abcam, Cambridge, MA, USA), rabbit anti-cyclophilin A (Santa Cruz Biotechnology, sc-20360-R) and mouse anti-HA (Merck), rabbit anti-STAT1 (Santa Cruz Biotechnology, sc-345), mouse anti-TNPO3 (Abcam), rabbit anti-RanBP2 (Thermo Fisher Scientific, PA1-O82), rabbit anti-TRIM34 (RNF21/IFP1, Abcam), and rabbit anti-TRIM5α (gift from P. Bieniasz, The Rockefeller University, New York, NY, USA) [52]. Secondary antibodies were goat anti-mouse or anti-rabbit horseradish peroxidase conjugates (GE Healthcare, Chicago, IL, USA) and goat anti-mouse AF555 (Thermo Fisher Scientific).

Debio 025 was purchased from Debiopharm (Lausanne, Switzerland) and was used at 200 nM 2 h before HIV-1 transduction. Nevirapine (Merck) was used at 5 μM. IFN-α2, IFN-β, and IFN-γ were purchased from R&D Systems (Minneapolis, MN, USA) and were used on human cell lines at 1000 UI/mL. For murine cells (IFN), Universal type I IFN (R&D Systems) was used at 200 UI/mL.

### 2.5. Stable Isotope Labeling by Amino Acids in Cell Culture (SILAC)

HEK293T cells were cultured for two weeks in SILAC DMEM lacking L-lysine and L-arginine and supplemented with 1% penicillin-streptomycin, 10% heat-inactivated dialyzed FBS, and 200 µg/mL L-proline (all purchased from Thermo Fisher Scientific). The medium was supplemented with 84 µg/mL L-arginine and 146 µg/mL L-lysine (both from Thermo Fisher Scientific) for the light medium (K0 R0), while the same amounts of ^13^C_6_-Larginine and ^2^H_4_-L-lysine, or ^13^C_6_-^15^N_4_-L-arginine and ^13^C_6_-^15^N_2_-L-lysine (from Cambridge Isotope Laboratories, Tewksbury, MA, USA, kindly provided by Guillaume Bossis, IGMM, Montpellier, France) were added to the medium and heavy media, respectively. After complete labeling monitored by MS analysis, 5 × 10^6^ cells from each condition were seeded in 10 cm Petri dishes. Light-labeled cells were transfected with an empty plasmid, whereas medium- and heavy-labeled cells were transfected with plasmids expressing Daxx wt or 1-732 (ΔSIM) mutant, respectively. Cells were transduced 24 h post transfection with VSV-G pseudotyped HIV-1 at a MOI of 10. After 4 h, cell extracts were prepared and Daxx was immunoprecipitated using anti-HA antibodies.

### 2.6. Mass Spectrometry

Equal amounts of the three immunoprecipitation elutions (light, medium, and heavy) were mixed at a ratio 1:1:1. After denaturation at 95 °C during 5 min, proteins were separated by SDS-PAGE and stained with a protein staining solution (PageBlue, Thermo Fisher Scientific). Proteins were fractionated in three bands. Pieces of gel were excised and destained with three washes in 50% acetonitrile (ACN) and 50 mM triethylammonium (TEABC). After protein reduction (10 mM dithiothreitol in 100 mM TEABC at 60 °C for 30 min) and alkylation (55 mM iodoacetamide in TEABC at room temperature for 30 min in the dark), proteins were digested in-gel using Trypsin overnight (1 µg, Gold, Promega) as previously described [53]. Digest products were dehydrated in a vacuum.

The resulting peptide samples were resuspended in 10 µL of loading buffer (0.05% TFA and 2% ACN) and 2 µL were loaded onto an analytical 50 cm reversed-phase column (75 mm inner diameter, Acclaim Pepmap 100^®^ C18, Thermo Fisher Scientific). Peptides were separated with an Ultimate 3000 RSLC system (Thermo Fisher Scientific) coupled to a Q Exactive HF (Thermo Fisher Scientific) via a nano-electrospray source, using a 123 min gradient of 2 to 40% of buffer B (80% ACN, 0.1% formic acid) and a flow rate of 300 nL/min. MS/MS analyses were performed in a data-dependent mode. Full scans (375–1500 m/z) were acquired on the Orbitrap mass analyzer with a 60,000 resolution at 200 m/z. For the full scans, 3 × 10^6^ ions were accumulated within a maximum injection time of 60 ms and detected in the Orbitrap analyzer. The twelve most intense ions with charge states ≥2 were sequentially isolated to a target value of 1 × 10^5^ with a maximum injection time of 45 ms and fragmented by HCD (higher-energy collisional dissociation) in the collision cell (normalized collision energy of 28%) and detected in the Orbitrap analyzer at 30,000 resolution.

MS/MS data analysis was performed using Proteome Discoverer software (v2.4.0.305, Thermo Fisher Scientific). All MS/MS spectra were searched using the SequestHT search engine against the UniProtKB Reference proteome UP000005640 database for Homo sapiens (release 2019_11, https://www.uniprot.org/), sequences of mutant Daxx protein and viral proteins, and a homemade contaminant database. Carbamidomethylation was set as fixed cysteine modification. Dynamic modifications were defined as methionine oxidation, N-terminal acetylation, methionine loss, and SILAC 3plex labeling (Arg0 Lys0/Arg6 Lys4/Arg10 Lys8). False discovery rate (FDR) for proteins was set to 1% for high confidence and 5% for medium confidence identification. A representative protein ID in each protein group (Protein Group IDs column) was automatically selected using an in-house bioinformatics tool (Master-Leading v3.2ML1) as the best annotated protein in UniProtKB (reviewed entries rather than automatic ones). Graphical representation and analysis of quantification data were performed using Perseus (v1.6.10.43) [54]. Abundance ratio based on median peptides ratios was used to highlight proteins differentially expressed between conditions. An outlier significance score for log abundance ratios was calculated (Significance A) and a FDR was calculated using a two-sided test (Benjamini–Hochberg FDR) [55].

### 2.7. Immunoprecipitations

Cells were lysed in immunoprecipitation (IP) lysis buffer containing 50 mM Tris-HCl (pH7.4), 150 mM NaCl, 1 mM EDTA, 1% Triton X-100, EDTA-free protease inhibitor cocktail (Merck), and 10 mM iodoacetamide (Merck). Cell lysates were incubated overnight at 4 °C with monoclonal anti-HA Agarose beads (Merck) or with anti-Daxx antibodies and Protein G Sepharose beads (Thermo Fisher Scientific). Beads were washed three times in lysis buffer and eluted with 2× SDS loading buffer at 95 °C. For immunoblotting, proteins were resolved by SDS-PAGE and transferred onto a nitrocellulose membrane.

### 2.8. Immunofluorescence Microscopy

Cells were grown on 12 mm round coverslips and fixed with 4% paraformaldehyde for 15 min, rinsed in PBS, incubated in NH4Cl 50 mM for 10 min, permeabilized with 0.5% Triton X100 for 15 min and blocked with 0.5% BSA/2% Normal Goat Serum for 10 min. Then, cells were incubated with primary antibodies at room temperature for 30 min. Slides were rinsed in PBS 0.5% BSA and incubated at room temperature in the dark for 30 min with secondary antibodies and rinsed in PBS 0.5% BSA. Finally, slides were washed in PBS, counterstained with Hoechst 33,342, and mounted in Fluoromount-G medium (Thermo Fisher Scientific). Images were digitally acquired with a Zeiss LSM 710 confocal microscope (Carl Zeiss, Jena, Germany).

### 2.9. Fate-of-Capsid Assays

The HIV-1 core stability in cells was assessed by the separation of soluble CA from CA cores on a sucrose cushion, using the fate-of-capsid assay [56]. At 4 h post transduction, the cells were lysed using hypotonic lysis buffer and a dounce homogenizer and briefly centrifuged to clarify the cytosol fractions. Then, the lysates were layered onto a 50% sucrose cushion and ultracentrifuged at 100,000× *g* for 2 h. The soluble CA (top 100 μL fraction) and intact cores (pellet) were analyzed by Western blotting of the CA (p24) signal. For the identification of proteins associated with incoming viral cores, we followed the same experimental procedure, except that intact HIV-1 cores were pulled down 2 h post transduction, and only the pellet fractions were analyzed by Western blot.

### 2.10. RT-QPCR Analyses

Total RNA was extracted using a RNeasy Mini kit and was submitted to DNase treatment (Qiagen), following the manufacturer’s instructions. RNA concentration and purity were evaluated by spectrophotometry (NanoDrop 2000c, Thermo Fisher Scientific). In addition, 500 ng of RNA were reverse transcribed with both oligo dT and random primers, using a PrimeScript RT Reagent Kit (Perfect Real Time, Takara Bio Inc., Kusatsu, Japan) in a 10 μL reaction. Real-time PCR reactions were performed in duplicate using Takyon ROX SYBR MasterMix blue dTTP (Eurogentec, Liège, Belgium) on an Applied Biosystems QuantStudio 5 (Thermo Fisher Scientific). Transcripts were quantified using the following program: 3 min at 95 °C followed by 35 cycles of 15 s at 95 °C, 20 s at 60 °C, and 20 s at 72 °C. Values for each transcript were normalized to expression levels of RPL13A (60S ribosomal protein L13a), using the 2-ΔΔCt method. Primers used for quantification of transcripts by real-time quantitative PCR are indicated below:

- RPL13A (human) forward primer, 5′-AACAGCTCATGAGGCTACGG-3′ and reverse primer, 5′-TGGGTCTTGAGGACCTCTGT-3′;
- Daxx (human) forward primer, 5′-TGCTGGATGATGATGACGAAGAT-3′ and reverse primer, 5′-CTCAGAGGAGCTAGGGGCTTC-3′;
- PML (human) forward primer, 5′-ATCACCCAGGGGAAAGATGC-3′ and reverse primer, 5′-TGAACCTGGGCCTTCACTCT-3′;
- IFIT1 (human) forward primer, 5′-AGGACAGGAAGCTGAAGGAG-3′ and reverse primer, 5′-AGTGGGTGTTTCCTGCAAGG-3′;
- STAT1 (human) forward primer, 5′-CAGAGCCAATGGAACTTGATGG-3′ and reverse primer, 5′-TCCGAGACACCTCGTCAAACTC-3′;
- RPL13A (murine) forward primer, 5′-TGCTGCTCTCAAGGTTGTTCG-3′ and reverse primer, 5′-GCATCTTGGCCTTTTCCTTCC-3′;
- Daxx (murine) forward primer, 5′-CCCACATCGCCTTCAGACTT-3′ and reverse primer, 5′-AGTCCTCTCTTTTGCTGCCC-3′;
- PML (murine) forward primer, 5′-TCTGCGCAAGGCACTCTGTAG-3′ and reverse primer, 5′-TCTGGGTGATGCAGGAGATGA-3′;
- IFIT1 (murine) forward primer, 5′-TCCGTAGGAAACATCGCGTAG-3′ and reverse primer, 5′-TGTTGCTTGTAGCAGAGCCC-3′.

### 2.11. Quantification of HIV-1 Reverse Transcripts by QPCR

For real-time PCR experiments, 12-well plates were seeded with 1.5 × 10^5^ cells per well. The next day, cells were transduced with VSV-G pseudotyped HIV-1 pre-treated with 250 U/mL Benzonase nuclease (Merck) for 20 min at 37 °C. Cells were collected at various timepoints after addition of the virus and total DNA was extracted using DNeasy Blood & Tissue Kit (Qiagen). Real-time PCR reactions were performed in duplicate using Takyon ROX SYBR MasterMix blue dTTP (Eurogentec) on an Applied Biosystems QuantStudio 5. Transcripts were quantified using the following program: 3 min at 95 °C followed by 35 cycles of 15 s at 95 °C, 20 s at 60 °C, and 20 s at 72 °C. Values for each transcript were normalized to expression levels of GAPDH transcripts. The qPCR analysis of HIV-1 early reverse transcription (RT) products was carried out using M667/AA55M primers specific for minus-strand strong stop DNA, whereas late RT products were detected using M667/M661 primers, which amplify the RU5-primer binding site 5′ noncoding region, as previously described [40]. Primer sequences were as follows: GAPDH-F, 5′-ACTTCAACAGCGACACCCACT-3′; GAPDH-R, 5′-GTGGTCCAGGGGTCTTACTCC-3′; M667, 5′-GGCTAACTAGGGAACCCACTG-3′; AA55M, 5′-GCTAGAGATTTTCCACACTGACTAA-3′; and M661, 5′-CCTGCGTCGAGAGAGCTCCTCTGG-3′.

## 3. Results

### 3.1. Daxx Is a Mediator of the Type I IFN-Induced Inhibition of HIV-1 Reverse Transcription

**Figure 1:**
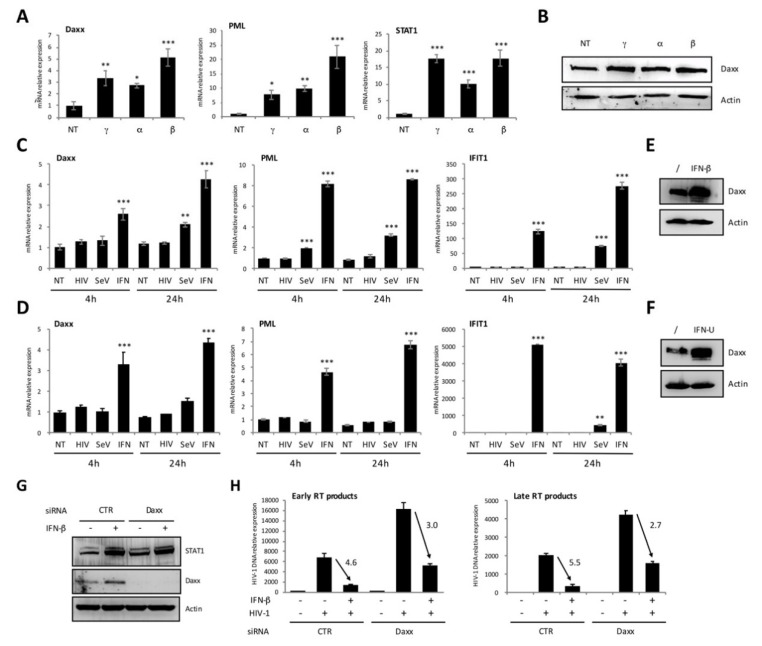
Death domain-associated protein 6 (Daxx) expression is induced by type I and type II interferons (IFNs) and inhibits HIV-1 reverse transcription. (**A**,**B**) HeLa cells were treated for 24 h with 1000 IU of IFN-α2, -β, or -γ and the levels of PML and STAT1 expression were assessed by RT-qPCR (**A**), whereas Daxx expression was determined by both RT-qPCR (**A**) and Western blot (**B**); (**C**,**D**) HeLa (C) or MDTF (D) cells were either treated with 1000 IU of IFN-β, transduced with VSV-pseudotyped HIV-1 (MO1 = 1), or infected with SeV (50 HAU/mL). After 4 or 24 h, the expression levels of Daxx, PML, and IFIT1 were determined by RT-qPCR; (**E**,**F**) HeLa cells were treated with 1000 IU of IFN-β (E) and MDTF were treated with 200 IU of Universal type I IFN (IFN-U) (F) and the expression of Daxx was evaluated by Western blot 24 h post treatment; (**G**,**H**) HeLa cells were transfected with irrelevant (CTR) or Daxx-specific siRNA and treated 24 h later with 1000 IU of IFN-β. After 24 h, the expression levels of Daxx and STAT1 were determined by Western blot (G). Then, cells were transduced for 4 h with VSV-G pseudotyped HIV-1 at a MOI of 1, and early and late RT products were quantified by qPCR. The IFN-induced inhibition folds in cells transfected with the CTR or the Daxx-specific siRNA are shown on the graphs (H). All data correspond to means ± SD of three independent experiments. * *p* < 0.05, ** *p* < 0.01, and *** *p* < 0.001 as determined by one-way ANOVA with Bonferroni post hoc test (A, C, D).

We previously reported that the Daxx protein inhibits HIV-1 replication by interfering with the reverse transcription step [40]. Here, we first investigated whether Daxx was an Interferon-stimulated gene (ISG) product and whether it participated to the type I IFN-induced inhibition of HIV-1 reverse transcription [57,58]. Although a few studies reported that the expression of Daxx mRNA or protein is upregulated by type I IFN in certain murine or human cells, it is still unclear whether Daxx constitutes a bona fide ISG product [49,59]. To answer this question, first, we quantified Daxx mRNA in HeLa cells treated with type I (IFN-α2 or IFN-β) or type II (IFN-γ) IFN for 24 h. As shown in Figure 1A, although its induction was rather modest as compared with two well-known ISGs such as PML and STAT1, Daxx expression was significantly enhanced by both type I and type II IFNs, and IFN-β was the most potent, leading to a five times enhancement of Daxx mRNA expression. These results were confirmed at the protein level (Figure 1B). Next, we investigated whether the expression of Daxx was also upregulated upon viral infections. We used HeLa and MDTF cells as models of human and murine cells, respectively. Cells were either transduced with a VSV-G pseudotyped HIV-1 vector, or infected with Sendai virus (SeV) for four or 24 h, and the expression of Daxx was quantified both at the mRNA (Figure 1C,D) and the protein level (Figure 1E,F). As a control, HeLa cells were treated by IFN-β, whereas MDTF cells were treated with Universal type I IFN, and both induced a potent enhancement of Daxx protein expression, as shown in Figure 1E (HeLa cells) and Figure 1F (MDTF cells). In both cell types, only SeV triggered a detectable induction of Daxx expression 24 h post infection, but less efficiently than the direct stimulation of cells with IFN (Figure 1C,D).

Since it is well established that HIV-1 reverse transcription is inhibited in cells treated with type I IFN [57,58], next, we investigated whether Daxx constituted a mediator of this IFN-induced block. For this purpose, we transfected cells with irrelevant or Daxx-specific siRNA and treated them, or not, with IFN-β before transduction with VSV-G pseudotyped HIV-1. The efficacy of Daxx silencing and the capacity of IFN-β to induce ISG expression were ascertained by Western blot using Daxx and STAT1 antibodies, respectively (Figure 1G). The amount of early and late reverse transcription products was quantified at 4 h post transduction by qPCR. Silencing of Daxx increased the detection of RT products, as previously shown [40]. As shown in Figure 1H, IFN treatment inhibited HIV-1 reverse transcription nearly five-fold, whereas Daxx silencing reduced this inhibition by 50% (Figure 1H), thus demonstrating that Daxx is one of the ISG contributing to the type I IFN-induced inhibition of HIV-1 reverse transcription.

### 3.2. The Capacity of Daxx to Interfere with HIV-1 Reverse Transcription Is Dependent on Its SIM Domain

Daxx is a scaffold protein that has been implicated in several biological processes, including apoptosis, cell survival, chromatin remodeling, gene regulation, antiviral defense, or DNA repair, (reviewed in [41,42,43,60]). The pleiotropic functions of Daxx are due to its capacity to interact with and to connect many different proteins in the cytoplasm and in the nucleus. The identification of a SUMO-interacting motif (SIM) at the C-terminus of Daxx was an important breakthrough in the understanding of Daxx’s interaction network [44]. Indeed, almost all Daxx partners are SUMOylated proteins and Daxx interacts with them through its SIM domain. Therefore, this domain is crucial for most Daxx functions and its capability to bind SUMO also controls Daxx SUMOylation [44].

**Figure 2:**
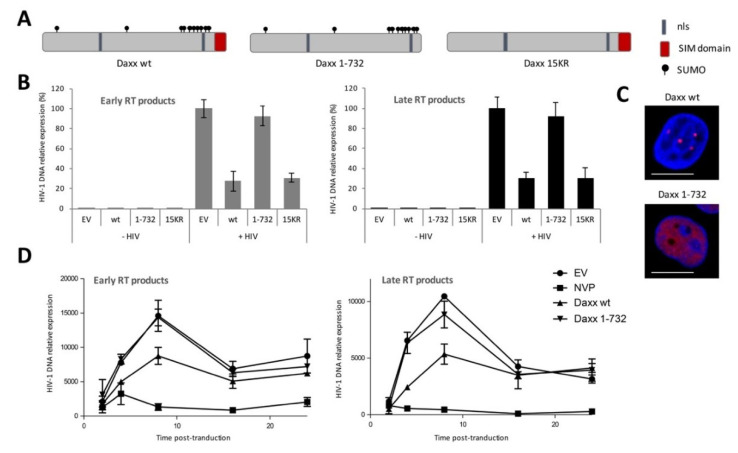
The inhibition of HIV-1 reverse transcription by Daxx requires its SUMO-interacting motif (SIM) domain. (**A**) Schematic representation of the Daxx wt protein and the 1-732 (ΔSIM) and 15KR mutants described in [44]; (**B**) HeLa cells were transfected with an empty vector (EV) or with plasmids encoding HA-tagged wt Daxx or 1-732 or 15KR Daxx mutants. Forty-eight hours post transfection, cells were transduced for 4 h with VSV-G pseudotyped HIV-1 at a MOI of 1, and early and late RT products were quantified by qPCR. The number of transcripts in the condition EV + HIV was arbitrarily set to 100%. Data represent the mean ± SD of one experiment performed in triplicate, representative of three independent experiments; (**C**) The expression of HA-tagged Daxx wt and 1-732 mutant was investigated by immunofluorescent confocal microscopy using anti-HA antibodies in HeLa cells. Scale bar = 2 μm; (**D**) HEK293T cells were transfected with empty vector (EV) or with plasmids encoding Daxx (wt or 1-732). Twenty-four hours post transfection, cells were treated or not with nevirapine (NVP), as indicated, and transduced with VSV-G pseudotyped HIV-1 at a MOI of 1 for 2 to 24 h. Following DNA extraction, early and late HIV-1 RT products were quantified by qPCR. Data represent the mean ± SD of one experiment performed in triplicate, representative of two independent experiments.

On the basis of this knowledge, we included the following two Daxx mutants in our study: one unSUMOylable Daxx mutant, in which 15 lysines have been mutated to arginines (15KR mutant) and a ΔSIM mutant (Daxx 1-732) lacking the C-terminal SUMO-interacting motif [44] (Figure 2A). In order to determine whether Daxx SUMOylation or the Daxx SIM domain were involved in its antiviral activity, we tested the capacity of these mutants to inhibit HIV-1 reverse transcription.

Interestingly, whereas the 15KR Daxx mutant was as efficient as the wt protein to inhibit HIV-1 reverse transcription, the ΔSIM mutant was no longer active (Figure 2B). This indicates that Daxx’s own SUMOylation status is not important for its antiviral activity, whereas its SIM domain is strictly required. This SIM domain allows Daxx to interact with its SUMOylated partners, as illustrated by the fact that, unlike the wt protein which is mainly localized within nuclear punctate structures typical of PML nuclear bodies [44,45], the ΔSIM mutant, which can no longer be recruited by SUMOylated PML, is uniformly expressed in the nucleoplasm (Figure 2C). Therefore, these results suggested that Daxx targets a SUMOylated protein, either viral or cellular, to inhibit HIV-1 reverse transcription.

Next, we performed kinetics experiments in order to better characterize the SIM-dependent effect of Daxx on HIV-1 reverse transcription. In particular, we set out to determine whether Daxx delays or blocks reverse transcription, and whether the ΔSIM mutant was inactive during the whole process. Thus, we overexpressed Daxx wt or ΔSIM mutant in HEK293T cells, transduced them with VSV-G pseudotyped HIV-1, and followed the synthesis of RT products over time by qPCR. As shown in Figure 2D, the peak of reverse transcription products occurs at 9 h post tranduction in cells transfected with an empty plasmid, and then gradually decreases. As anticipated, treatment with a RT inhibitor (nevirapine) completely abolished reverse transcription (Figure 2D). Results obtained with wt Daxx and the ΔSIM Daxx mutant showed that Daxx inhibits rather than delays reverse transcription and confirmed that the SIM domain of Daxx is necessary for its anti-HIV activity. The fact that Daxx affects the efficacy but not the kinetics of HIV-1 DNA synthesis suggests that Daxx-mediated block is irreversible.

### 3.3. Daxx Interacts with a Multiprotein Complex Containing CypA, TRIM5α, and HIV-1 CA

**Figure 3:**
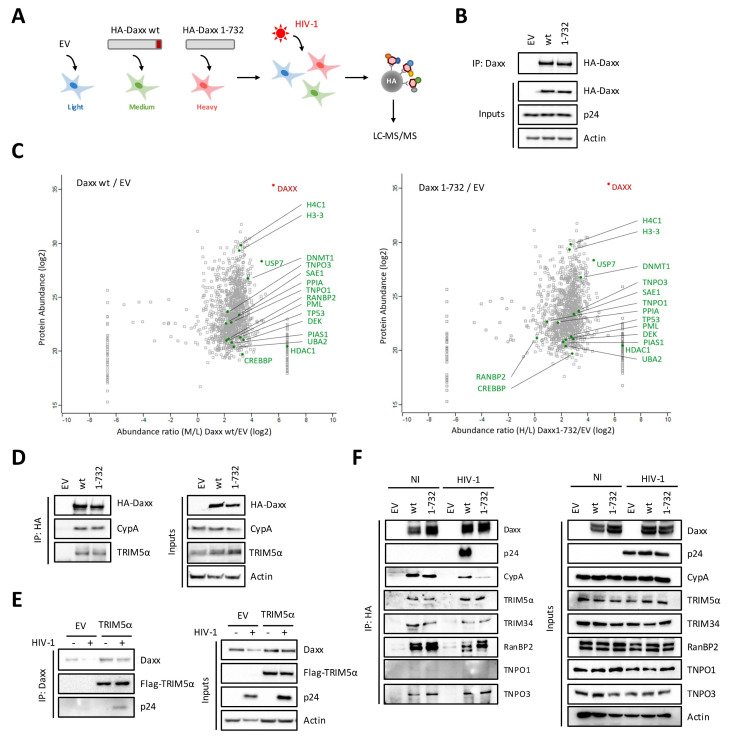
Identification of proteins interacting with Daxx by SILAC. (**A**) Schematic representation of the experimental procedure. HEK293T cells were cultured in medium containing light, medium, or heavy isotopes for 30 days. Then, cells were transfected with empty vector (EV) or with plasmids encoding HA-tagged Daxx wt or 1-732 (ΔSIM mutant). After 24 h, cells were transduced with VSV-G pseudotyped HIV-1 at a MOI of 10. At 4 h post transduction, Daxx was immunoprecipitated using anti-HA antibodies and the eluates were mixed in 1:1:1 ratio and subjected to proteomics experiments; (**B**) A fraction of cell extracts was kept for analysis by Western blot in order to evaluate the amount of Daxx and HIV-1 p24 in cell extracts; (**C**) Identification of Daxx interacting partners by SILAC. Graphs show the abundance ratios of proteins in cells expressing Daxx wt (left) or Daxx 1-732 (right) as compared with control cells (EV); (**D**) HEK293T cells were transfected with empty vector (EV) or with plasmids encoding HA-tagged wt Daxx or the 1-732 Daxx mutant. Following Daxx pull-down using anti-HA antibodies, the presence of HA-Daxx and endogenous cyclophilin A (CypA) and TRIM5α was assessed by Western blot; (**E**) HEK293T cells were transfected with an empty vector (EV) or a Flag-tagged hTRIM5α-expressing plasmid. Forty-eight hours post transfection, cells were transduced with VSV-G pseudotyped HIV-1 at a MOI of 10 for 4 h. Following Daxx pull-down using anti-Daxx antibodies, the presence of Flag-TRIM5α, HIV-1 p24, and endogenous Daxx was evaluated by Western blot; (**F**) HEK293T cells were transfected with empty vector (EV) or with plasmids encoding HA-tagged wt Daxx or the 1-732 Daxx mutant and were transduced, or not, 24 h later with VSV-G pseudotyped HIV-1 at a MOI of 10 for 4 h. Following Daxx pull-down using anti-HA antibodies, the presence of HA-Daxx, HIV p24, and endogenous CypA, TRIM5α, TRIM34, RanBP2, TNPO1, and TNPO3 was assessed by Western blot. Panels (D–F) show typical results representative of at least two independent experiments.

Although we determined that Daxx targeted a SUMOylated protein in order to interfere with HIV-1 reverse transcription, our early findings suggested that it was unlikely to be a viral protein. Therefore, we undertook to identify the cellular proteins that Daxx binds to in HIV-1 infected cells. For this, we used stable-isotope labeling by amino acids in cell culture (SILAC)-based quantitative proteomics, coupled with liquid chromatography tandem mass spectrometry (LC-MS/MS). As templates, we used HEK293T cells transfected with an empty plasmid or with HA-tagged wt or ΔSIM Daxx-expressing plasmids and transduced for 4 h with VSV-G pseudotyped HIV-1. Finally, we performed HA-immunoprecipitations and eluates were processed for mass spectrometry analysis (Figure 3A), after having evaluated the transfections, transduction and IP efficiencies by Western blot (Figure 3B). As shown in Figure 3C and Table 1, Daxx was one of the most abundant recovered proteins, as expected. Among the other most abundant proteins identified by SILAC, well-characterized Daxx partners, such as histone deacetylase 1 (HDAC1) [61], histones H4 and H3.3 [62], PML [45], or p53 [63], were also identified, thereby validating our approach (Figure 3C and Table 1). Surprisingly, SUMO was not identified as an interacting protein, although several enzymes implicated in the SUMOylation machinery were co-immunoprecipitated, including SUMO-interacting enzyme subunits 1 and 2, and the E3 SUMO-protein ligases PIAS1 and RanBP2 (Figure 3C and Table 1).

Interestingly, among the proteins interacting with Daxx, we identified several proteins that are known to bind HIV-1 capsids and to be involved in the early steps of HIV-1 replication, including TNPO1 [23], RanBP2 [64], TNPO3 [24], and CypA [22] (Figure 3C and Table 1). Among these, only RanBP2 has been shown to be SUMOylated [65], however, its main localization within nuclear pores in interphasic cells made us hypothesize it was unlikely to bind to incoming cores.

Since several CA binding proteins were strong hits in our proteomic analysis, we hypothesized that Daxx could engage incoming CA within a complex that contains several CA binding proteins, as well as SUMOylated proteins. Since TRIM5α is a well-established CA binding partner, competing with CypA for binding to CA [38,39], and is also known to be SUMOylated [66,67], it represents a potential candidate protein to be recruited by Daxx. In order to test this hypothesis, we transfected cells with HA-tagged Daxx wt or ΔSIM and immunoprecipitated Daxx using HA-antibodies. Endogenous CypA was pulled down, thus confirming the results obtained by the SILAC approach, as well as TRIM5α (Figure 3D).

In order to confirm these observations, we repeated the IP experiment but this time we overexpressed Flag-tagged TRIM5α instead of Daxx and performed the experiment in cells transduced, or not, with VSV-G pseudotyped HIV-1 for 4 h. Following endogenous Daxx IP, we found an interaction between TRIM5α, Daxx, and HIV-1 CA (p24) in transduced cells (Figure 3E), further suggesting that Daxx resides within a complex that also contains TRIM5α and CypA, supposedly organized around incoming HIV-1 cores.

In an attempt to better characterize this complex, cells were transfected with HA-tagged Daxx wt or ΔSIM, transduced or not with VSV-G pseudotyped HIV-1 for 4 h and the proteins in complex with Daxx or the ΔSIM mutant were identified by Western blot following IP with anti-HA antibodies (Figure 3F). Independently of HIV-1, Daxx associated with CypA, TRIM5α, TRIM34, RanBP2, and TNPO3, but not with TNPO1. Strikingly, whereas both Daxx wt and ΔSIM interacted with TRIM5α, TRIM34, RanBP2, and TNPO3, only Daxx wt was found to associate with HIV-1 capsid and CypA in transduced cells (Figure 3F). This result strongly suggests that Daxx is in a complex with HIV-1 cores and CypA in a SIM-dependent interaction. The lack of SIM domain prevented Daxx from binding to CypA and HIV-1 capsid, and therefore explains why the ΔSIM mutant is devoid of antiviral activity.

### 3.4. Daxx Associates to Incoming HIV-1 Cores and Interferes with Uncoating

**Figure 4:**
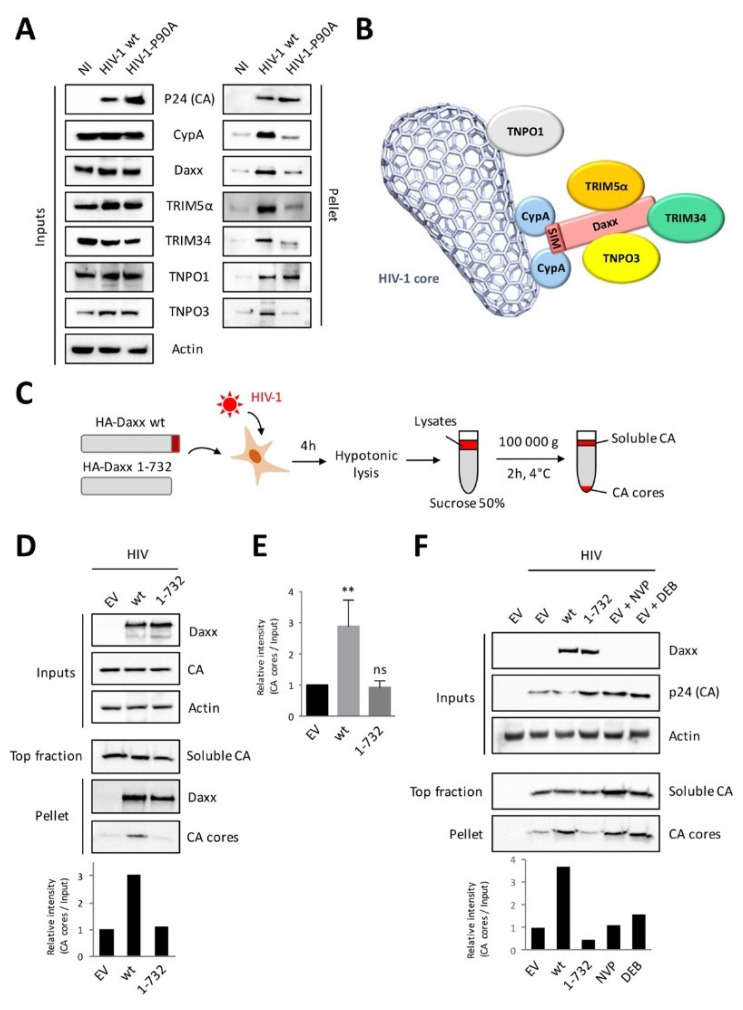
Daxx associated to incoming HIV-1 cores and interferes with their uncoating in a SIM-dependent manner. (**A**) HEK293T cells were transduced with VSV-G pseudotyped HIV-1 wt or P90A at a MOI of 10 for 2 h. Cells were lysed using hypotonic lysis buffer and a dounce homogenizer, and then the lysates were layered onto a 50% sucrose cushion and ultracentrifuged at 100,000× *g* for 2 h, in order to pull down intact cores (recovered in the pellet). Equal amounts of HIV-1 cores were analyzed by Western blot in order to evaluate the presence of p24 (CA), CypA, Daxx, TRIM5α, TRIM34, TNPO1, and TNPO3; (**B**) This cartoon represents a model where Daxx interacts with CypA bound to incoming HIV-1 capsids and coordinates the formation of a multiprotein complex containing TRIM5α, TRIM34, and TNPO3. TNPO1, which is not within this complex, binds directly to the viral core [23]; (**C**) Schematic representation of fate-of-capsid assay; (**D**) HEK293T cells were transfected with empty vector (EV) or with plasmids encoding HA-tagged Daxx wt or the 1-732 Daxx mutant. Twenty-four hours post transfection, cells were transduced with VSV-G pseudotyped HIV-1 at a MOI of 10 for 4 h. Cells extracts were prepared as in (A) and ultracentrifuged at 100,000× *g* for 2 h on a 50% sucrose cushion, in order to separate soluble CA (top fraction) from intact cores (pellet). Both fractions were analyzed by Western blot using anti-p24 and Daxx antibodies. Quantification of band intensity shows the amount of CA cores relative to inputs (EV set to 1); (**E**) This graph shows the mean band intensity (amount of CA cores relative to inputs) ±SD of three independent experiments. ** *p* < 0.01; ns, non-significant, as determined by one-way ANOVA with Bonferroni post hoc test; (**F**) The same experimental procedure as in panel (D) was followed, but some cells transfected with EV were treated with nevirapine (NVP) or Debio 025 (DEB025) before HIV-1 transduction. Quantification of band intensity shows the amount of CA cores relative to inputs (EV set to 1).

Since we established that Daxx binds to CypA and HIV-1 capsid in a SIM-dependent manner, and could also interact with TRIM5α, RanBP2, TNPO3, and TRIM34, next, we sought to investigate whether a multiprotein complex containing Daxx formed on incoming HIV-1 cores. Moreover, since our results showed that CA and CypA were the critical determinants in binding to the SIM domain of Daxx, we hypothesized that Daxx associated with incoming cores by binding to CA-bound CypA and recruited additional proteins. If this is true, Daxx should not be able to bind to HIV-1 cores that are devoid of CypA, such as the CypA-binding deficient HIV-1 mutant P90A [33]. To test this hypothesis, we transduced cells for 2 h with VSV-G pseudotyped HIV-1 wt or P90A and intact viral cores were pulled down by ultracentrifugation on a sucrose cushion (Figure 4A). As anticipated, CypA, Daxx, TRIM5α, TRIM34, TNPO1, and TNPO3 were recovered in the pellet fraction of HIV-1 wt infected cells, associated with intact capsids. With the exception of TNPO1, which has been previously shown to bind P90A CA [23], these were almost absent from the pellet in cells infected with the P90A capsid mutant (Figure 4A). We had already demonstrated that Daxx interfered with HIV-1 reverse transcription and that its SIM domain was required for this activity. Now, our results demonstrate that Daxx binds to incoming HIV-1 cores in a CypA-dependent manner and suggest that it resides within a complex together with TRIM5α, TRIM34 and TNPO3 (Figure 4B). Interestingly, unlike Daxx and its interacting partners, TNPO1 bound to HIV-1 cores independently of the presence of CypA, in agreement with a recent study [23]. This confirms that TNPO1 binds directly to HIV-1 capsid and does not reside within the CypA/Daxx multiprotein complex. Concerning RanBP2, we failed to detect its presence in this HIV-1 capsid pull-down assay, thus suggesting it is not part of the CypA/Daxx complex. Therefore, it is possible that Daxx and RanBP2 interact independently of HIV-1 infection, possibly through the C-terminal cyclophilin domain of RanBP2 [68].

The Daxx-associated multiprotein complexes assemble onto viral cores during the first steps of HIV-1 replication following viral entry, when reverse transcription, but also uncoating, occur. Since CypA [34,37,69,70], TRIM5α [14], TNPO3 [37], and TRIM34 [28] have key roles in HIV-1 core integrity, we sought to examine the impact of Daxx expression on HIV-1 uncoating. To address this question, the stability of viral cores was assessed in cells transfected with Daxx wt or ΔSIM and transduced for 4 h with VSV-G pseudotyped HIV-1 using the fate-of-capsid assay (Figure 4C). The cytosol of transduced cells was layered onto a sucrose cushion, after which ultracentrifugation yielded soluble CA in the uppermost fraction and CA cores in the pellet, as previously described [56] (Figure 4C). As shown in Figure 4D,E, whereas a small proportion of CA was recovered in the pellet of empty plasmid- (EV) or Daxx ΔSIM-expressing cells, the amount of intact cores was significantly increased (three-fold) in cells overexpressing wt Daxx, suggesting that Daxx stabilizes HIV-1 cores.

In another set of experiments, we added two conditions where EV-transfected cells were treated with nevirapine or Debio-025, a cyclosporin A analogue that displaces CypA from HIV-1 cores. Once again, the amount of intact HIV-1 cores in wt Daxx-expressing cells was more than three times higher than that in the control cells or in cells expressing the ΔSIM Daxx mutant (Figure 4F), whereas the proportion of HIV-1 CA in the pellet was not much affected by nevirapine or by Debio-025. Altogether, our results are in favor of a model in which Daxx stabilizes incoming HIV-1 cores in a SIM-dependent manner, through the binding of Daxx to CA-bound CypA and the recruitment of other proteins including TRIM5α, TNPO3, and TRIM34, altogether increasing the stability of viral cores (Figure 4B).

## 4. Discussion

While hijacking many cellular proteins to achieve its replication, HIV-1 is also the target of other proteins expressed by host cells in order to limit viral propagation. These factors, known as restriction factors, inhibit HIV-1 at multiple stages of its replication cycle. Some of these antiviral factors are constitutively expressed by host cells, and therefore constitute a first line of defense. These defenses are referred to as intrinsic immunity, which is considered as a third arm of immunity, in addition to the traditionally bipartite immune system of innate and adaptive immunity [1]. Some others are upregulated by type I IFN, and thus are part of innate defenses. Among these, APOBEC3G, TRIM5α, tetherin, and MxB have been reported to be potent IFN-induced anti-HIV factors [8,9,71,72,73,74].

In 2015, we identified Daxx as a new cellular protein that interferes with HIV-1 replication and identified reverse transcription as the step affected [40]. In the present study, we show that like most restriction factors, Daxx expression is upregulated by type I IFNs. Importantly, we also identified Daxx as one of the mediators of the anti-HIV activity triggered by type I IFNs.

Using Daxx mutants, we demonstrated that Daxx needs its SIM domain to inhibit HIV-1 reverse transcription, thus suggesting that it interferes with this step of viral replication by targeting a SUMOylated viral or cellular protein. Two HIV-1 proteins have been shown to be SUMO substrates, p6 [75] and integrase (IN) [76]. We rapidly ruled out the hypothesis that Daxx could access HIV-1 integrase, since it is associated with viral RNA and confined inside capsid cores until they reach the nucleus [17]. However, it is interesting to note that Daxx could interact with retroviral integrases, including that of HIV-1, and was found to inhibit proviral expression, thus suggesting that Daxx could also target HIV-1 in the nucleus after nuclear transport [48,77]. The case of p6 is more puzzling, since in the study by Gurer C et al. demonstrating that HIV-1 p6 was covalently conjugated to SUMO1, they also identified Daxx as a protein interacting with p6 in a SUMO-dependent manner [75]. As such, we performed preliminary experiments to confirm this interaction and the absence of any described role for p6 in the early steps of HIV-1 infection prompted us to perform a global proteomic screen to identify cellular targets of Daxx in HIV-1 infected cells. Using LC-MS/MS, we identified many known partners of Daxx, such as histone deacetylase 1 (HDAC1) [61], histones H4 and H3.3 [62], PML [45], or p53 [63], thus validating the screen. Interestingly, we also identified several well-characterized HIV-1 CA-interacting proteins, including TNPO1 [23], RanBP2 [64], TNPO3 [24], and CypA [22]. Given the central role that HIV-1 CA and its cellular partners play in the early steps of HIV-1 replication, including reverse transcription, we thought these proteins could be involved in the anti-HIV-1 activity of Daxx.

Using classical biochemical experiments, we confirmed that Daxx associates with CypA, as well as with HIV-1 CA in a SIM-dependent manner. If the interaction between the SIM domain of Daxx and CypA is direct, this could imply that CypA is SUMOylated, at least when bound to HIV-1 capsid, a hypothesis that needs to be addressed. Furthermore, we demonstrated that Daxx associates with incoming HIV-1 cores at 2 h post infection and resides within a complex containing CypA, TNPO3, TRIM5α, and its recently identified cofactor TRIM34 [28]. Whereas human TRIM5α was believed, until recently, not to have any restriction activity towards HIV-1, its counterparts expressed in old-world monkey are potent inhibitors of HIV-1 reverse transcription through their capacity to bind incoming capsids and trigger their uncoating [8,14]. However, it has recently been shown that human TRIM5α was also able to block HIV-1 in primary cells, but was counteracted by CypA, which protects incoming cores from its restriction [38,39]. Considering the prominent role that CypA, TNPO3, TRIM5α, and TRIM34 have on HIV-1 capsid stability [14,28,34,37,69,70], we sought to investigate the potential influence of Daxx on the cytoplasmic fate of incoming cores. Strikingly, the expression of Daxx induced a potent inhibition of HIV-1 uncoating. As with the inhibition of reverse transcription, the Daxx SIM domain was strictly required, since the ΔSIM Daxx mutant was unable to stabilize HIV-1 capsid. Therefore, our results reveal that Daxx is both able to inhibit HIV-1 reverse transcription and to prevent uncoating. Since these are two tightly interdependent processes that occur concomitantly during the first hours of HIV-1 replication, further studies are required to determine which event is the cause and which one is the consequence [15,16,18,19]. However, the timing and location of HIV-1 uncoating have been under debate for more than a decade, pointing out the difficulty to decipher these complex steps of HIV-1 replication [15,17,23,78].

Altogether, our results suggest that, through a SIM-dependent interaction with CA-bound CypA and the recruitment of a complex containing TRIM5α, TRIM34, and TNPO3, Daxx associates with and stabilizes HIV-1 cores, thus preventing uncoating and reverse transcription. This model fits with the known function of Daxx, which acts as a scaffold to bridge different proteins [41,42,43]. Interestingly, TNPO1, which was recently shown to orchestrate HIV-1 uncoating and nuclear import [23], was not present in Daxx’s complex. Unlike Daxx and its interacting partners, TNPO1 bound as efficiently to wt and P90A HIV-1 capsids, thus confirming that the association of TNPO1 with incoming HIV-1 cores is independent of the presence of CypA [23].

Interestingly, Daxx was recently identified in a CRISPR-based screen aiming to identify ISGs that inhibit HIV-1 infection in THP-1 cells [28]. This screen allowed the identification of genes whose knockout rescues the IFN-induced inhibition of viral replication, such as genes implicated in IFN signaling as well as restriction factors. Daxx was the 43rd highest-scoring gene identified in the screen, a lower rank than MxB, tetherin or TRIM5, but higher than other well-known anti-HIV ISGs, such as IFITM3 or TRIM22 [28]. Strikingly, unlike the other antiviral ISGs, Daxx was no longer found when the same screen was performed using the HIV-1 P90A capsid mutant [28], thus confirming that Daxx is an anti-HIV ISG and also reinforcing the pertinence of our model in which Daxx targets HIV-1 cores by binding to CypA.

On the whole, our study answers many questions regarding the molecular mechanism underlying Daxx-mediated HIV-1 restriction, but it also opens new routes of investigation. For instance, although we identified multiprotein complexes containing Daxx, CypA, TRIM5α, TRIM34, and TNPO3 that associate with incoming HIV-1 capsids, other potentially important factors could also be present and need to be identified. Among these proteins, we need to determine which ones are directly involved in the inhibition of uncoating or reverse transcription and which ones are dispensable.

In a previous work, we found that Daxx overexpression inhibited wt HIV-1 spreading infection in activated PBMCs [40]. However, since all experiments described in the present study were performed using VSV-G pseudotyped virus and immortalized cell lines, further studies are required to confirm our findings in a physiological model of HIV-1-infection in human primary cells.

Another important question remains to be addressed. Indeed, we previously showed that Daxx inhibited reverse transcription of HIV-1, and also of SIVmac and MLV, and was even able to interfere with the retrotransposition of endogenous retroviruses [40]. Since CypA does not bind SIVmac or MLV capsid, it is unlikely to participate in these Daxx-mediated antiviral activities [32]. TRIM5α, in contrast, could be involved, since it displayed a much broader activity and has even been shown to restrict endogenous retroviruses [79]. In any case, further studies are required in order to determine whether Daxx employs virus-specific or a common mechanism to interfere with retroviral infections.

## Figures and Tables

**Table 1:** Proteins interacting with Daxx wt or Daxx 1-732, identified using stable-isotope labeling by amino acids in cell culture (SILAC).

Protein Name	Symbol	Accession	Abundance Ratio (log2)		Abundance Ratio (log2) Significance A		# Peptides	# Unique Peptides
			Daxx wt/EV	Daxx 1-732/EV	Daxx wt/EV	Daxx 1-732/EV		
Death domain-associated protein 6	DAXX	Q9UER7	5.59	5.55	≤0.05	≤0.05	54	4
Histone deacetylase 1	HDAC1	Q13547	6.64	6.649	≤0.05	≤0.05	2	1
Ubiquitin carboxyl-terminal hydrolase 7	USP7	Q93009	4.73	4.45	≤0.05	≤0.05	55	55
DNA (cytosine-5)-methyltransferase 1	DNMT1	P26358	3.71	3.45	0.1583	0.2876	35	35
Protein DEK	DEK	P35659	3.40	2.91	0.3240	0.7041	2	2
CREB-binding protein	CREBBP	Q92793	3.33	2.81	0.3732	0.8001	3	1
Histone H4	H4C1	P62805	3.22	2.68	0.4595	0.9294	11	11
Cellular tumor antigen p53	TP53	P04637	3.16	2.73	0.5108	0.8793	4	4
Histone H3.3	H3-3A	P84243	3.08	2.61	0.5837	1.0000	6	4
Protein PML	PML	P29590	2.14	2.32	0.6071	0.8042	3	3
SUMO-activating enzyme subunit 1	SAE1	Q9UBE0	3.10	2.95	0.5651	0.6669	8	8
SUMO-activating enzyme subunit 2	UBA2	Q9UBT2	2.70	2.35	0.9781	0.8241	2	2
E3 SUMO-protein ligase PIAS1	PIAS1	O75925	2.53	2.13	0.8864	0.6816	2	2
E3 SUMO-protein ligase RanBP2	RANBP2	P49792	2.31	0.14	0.7246	≤0.05	4	2
Transportin-1	TNPO1	Q92973	2.50	0.92	0.8639	0.1486	6	6
E3 SUMO-protein ligase RanBP2	RANBP2	P49792	2.31	0.14	0.7246	≤0.05	4	2
Transportin-3	TNPO3	Q9Y5L0	2.23	3.29	0.6682	0.3894	8	8
Peptidyl-prolyl cis-trans isomerase A	PPIA	P62937	2.10	1.73	0.5807	0.4520	4	4

Proteins are ordered as follow: In red, Daxx; in green, known Daxx partners; in yellow, enzymes of the SUMO machinery; in blue, proteins known to interact with HIV-1 capsid. #: Number of.
